# Antioxidant and Hypoglycemic Potential of Essential Oils in Diabetes Mellitus and Its Complications

**DOI:** 10.3390/ijms242216501

**Published:** 2023-11-19

**Authors:** Simona Gabriela Bungau, Cosmin Mihai Vesa, Cristian Bustea, Anamaria Lavinia Purza, Delia Mirela Tit, Mihaela Cristina Brisc, Andrei-Flavius Radu

**Affiliations:** 1 Doctoral School of Biomedical Sciences, University of Oradea, 410087 Oradea, Romania; sbungau@uoradea.ro (S.G.B.); mirela_tit@yahoo.com (D.M.T.); andreiflavius.radu@uoradea.ro (A.-F.R.); 2 Department of Pharmacy, Faculty of Medicine and Pharmacy, University of Oradea, 410028 Oradea, Romania; purza_lavinia@yahoo.com; 3 Department of Preclinical Disciplines, Faculty of Medicine and Pharmacy, University of Oradea, 410073 Oradea, Romania; 4 Department of Surgery, Oradea County Emergency Clinical Hospital, 410169 Oradea, Romania; 5 Department of Medical Disciplines, Faculty of Medicine and Pharmacy, University of Oradea, 410073 Oradea, Romania; briscristina@yahoo.com

**Keywords:** antioxidants, essential oil, diabetes mellitus, phytochemistry, bibliometric analysis

## Abstract

Since the earliest times, essential oils (EOs) have been utilized for medicinal and traditional purposes. However, in recent decades, an increasing interest has developed due to the need to rediscover herbal remedies and adjuvant therapies for the management of various diseases, particularly chronic ones. The present narrative review examines the potential for EOs to exert hypoglycemic and antioxidant effects in diabetes mellitus, analyzing the main publications having evaluated plant species with potentially beneficial effects through their phytocompounds in diabetes mellitus and its complications. Numerous species have shown promising characteristics that can be used in diabetes management. The hypoglycemic effects of these EOs are attributed to their capacity to stimulate glucose uptake, suppress glucose production, and increase insulin sensitivity. Moreover, EOs can alleviate the oxidative stress by manifesting their antioxidant effects via a variety of mechanisms, including the scavenging of free radicals, the regulation of antioxidant enzymes, and the decreasing of lipid peroxidation, due to their diverse chemical composition. These findings demonstrate the possible benefits of EOs as adjuvant therapeutic agents in the management of diabetes and its complications. The use of EOs in the treatment of diabetes shows good potential for the development of natural and effective strategies to enhance the health outcomes of people with this chronic condition, but additional experimental endorsements are required.

## 1. Introduction

Essential oils (EOs) are liquid combinations of volatile substances resulting from the secondary metabolism of aromatic plants. Over 60 different plant families can produce the complex mixtures of compounds, which typically contain alcohols, aldehydes, esters, ethers, ketones, and terpenoids, in varying concentrations. The main constituents are monoterpenes and sesquiterpenes, along with, to a smaller degree, aromatic and aliphatic components [1].

EOs are produced by extracting the plant raw materials using a considerable number of techniques that have been optimized over time. The method of extraction chosen is crucial since it dictates the amount, kind, along with stereochemical structure of the resulting EO molecules. Given their therapeutic characteristics, EOs have been utilized for thousands of years in rituals and medicine [2].

Numerous studies have been conducted to determine the therapeutic effects of EO, the primary ingredient utilized in inhalation treatment. There is evidence that using EOs along with traditional medicine may effectively decrease anxiety and relieve pain [3].

The administration of EOs might take the form of inhalation, oral intake, or direct skin contact. Inhalation is probably the most widely used administration route. Lavender and bergamot EOs are the most frequently utilized for relaxing, either alone or in combination, with observed pharmacodynamic interactions. Furthermore, EOs from various plant extracts have been researched and shown to have varied effects [4].

Despite the fact that EOs have been investigated for over sixty years, there has been an increase in interest in recent years as people have become more interested in natural cures [2]. In the scientific literature, EOs have been evaluated for their potential utility in the management of complex pathologies [5,6], including diabetes mellitus (DM) [7,8,9] and obesity [10].

The prevalence of diabetes mellitus (DM) among adults aged 20–79 worldwide was predicted to reach 536.6 million by 2021 and 783.2 million by 2045, with over 90–95% of cases being type 2 DM (T2DM) and 5–10% being type 1 DM (T1DM). T2DM is more common in people aged 75–79, with incidence rates being identical in men and women. In 2021, the prevalence was predicted to be greater in urban compared to rural regions and high-income than low-income states. In comparison to high- and low-income countries, middle-income countries are predicted to experience the largest relative rise in the incidence of T2DM between 2021 and 2045. The costs associated with treating both types of DM worldwide have been predicted to USD 966 billion USD in 2021 and are expected to rise to 1054 billion USD by 2045. Currently more than 10.5% of the world’s population of adults suffer from T2DM or T1DM, resulting in little more than half a billion individuals affected globally [11].

Central obesity, hypertension, atherogenic dyslipidemia, and insulin (INS) resistance are components of the metabolic disease complex known as metabolic syndrome. These elements support a persistent inflammatory response, which promotes the emergence of cardiovascular disease (CVD). Metabolic syndrome has gained significant relevance as a result of rising obesity rates around the world; early detection and intervention are therefore relevant in reducing mortality rates [12].

T2DM or T1DM patients are more likely to develop complications such coronary heart disease, neuropathy, peripheral vascular disease, nephropathy, and retinopathy. Complications from T2DM may appear 10–20 years after the initial diagnosis, even though these may be the individual’s primary symptoms in those cases. Impairment to the blood arteries is one of the main long-term consequences. A person’s risk of developing CVD is almost doubled when suffering from T2DM or T1DM. Myocardial infarction, angina, peripheral vascular disease, and stroke are the most common *macro-vascular* pathologies [13]. Moreover, an additional prominent factor implicated as an underlying etiological factor for both types of DM is dysbiosis within the gut microbiota, characterized by an imbalance in the composition of intestinal microorganisms [14].

Medicines used in T2DM alter the metabolism of dietary carbohydrates, being frequently and commonly used in the management and therapy of this disease. These medications include peroxisome proliferator-activated receptor (PPAR) ligands, adenosine monophosphate-activated protein kinase (AMPK) activators, α-amylase, and α-glucosidase inhibitors. However, these medications have negative side effects that may be reduced by EOs that have antidiabetic properties [15].

In the context of T2DM management, it is noteworthy highlighting the beneficial role of diet. The ketogenic diet is one of the most widely used approaches and it is characterized by a dietary regimen that substitutes glucose with ketone bodies, and it has demonstrated efficacy in the management of various medical conditions, including metabolic disorders [16]. Functional foods high in polyphenols are currently being considered as potentially special supplemental and nutraceutical remedies for T2DM based on their biological advantages. A unique oral strategy to control glucose metabolism and hyperglycemia involves inhibiting α -glucosidase and α-amylase enzymes utilizing natural compounds, particularly polyphenols [17].

Although T1DM and T2DM have different pathophysiologies, some common features like beta cell dysfunction and apoptosis, increased oxidative stress, hyperlipidemia, and microvascular and macrovascular complications exist [18].

Plant extracts and plants are useful in reducing the impact of chronic glucose exposure and increasing the INS sensitivity of certain cells. EOs extracted from plants have mostly been investigated in the animal models of DM (e.g., streptozotocin-induced diabetes mellitus in rats) which one might think better resembles T1DM pathophysiology; however, beta-cell dysfunction and apoptosis are common in all diabetes patients, and therefore our review is relevant regardless of what type of DM is mentioned [19].

The aim of the present paper is to examine existing scientific literature and outline current knowledge regarding the potential benefits of several EOs for diabetes and its complications. The specific objective is to investigate the hypoglycemic effects of EOs, specifically their ability to stimulate glucose uptake, inhibit glucose production, and boost INS sensitivity. In addition, the antioxidant properties of EOs and their function in reducing oxidative stress, an important issue in diabetes and its associated complications, have been examined. This narrative review contributes to the scientific literature through its exhaustive investigation into the prospective benefits and mechanisms of action of EOs in the management of diabetes. It provides a valuable resource for investigators, healthcare professionals, and individuals interested in natural and alternative approaches to diabetes care by synthesizing and centralizing the available scientific data.

## 2. Impact of Essential Oils on Oxidative Stress and Enzymes in Diabetes Mellitus

Since oxidative stress is involved in the development of both types of DM, medications that reduce this type of stress may be useful in managing the disease. Numerous studies have been conducted to examine the effects of antioxidant therapy on both the avoidance and management of both types of DM complications in diabetic individuals and animal models of the disease [20].

Increased levels of free radicals suppress the nuclear transcription factors that regulate the expression of the INS gene such as pancreatic and duodenal homeobox 1 (Pdx-1), an INS promoter factor 1, and V-maf musculoaponeurotic fibrosarcoma oncogene homolog A (MafA), a transcription factor, thus lowering the production of INS at the messenger ribonucleic acid (mRNA) level [21].

**Figure 1:**
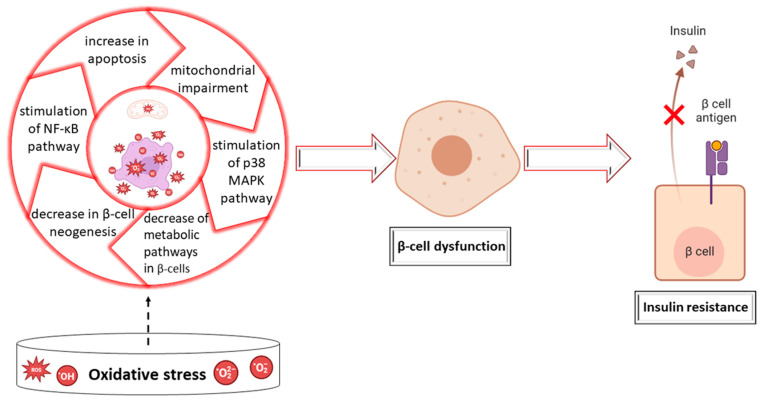
Potential molecular processes linking oxidative stress and dysfunction in beta cells that may contribute to diabetes mellitus development: nuclear factor kappa B (Nf-κB); mitogen-activated protein kinases (MAPK).

Multiple molecular processes are utilized by oxidative stress to impair beta-cell function. This significantly decreases the INS production, compromises the incorporation of proINS vesicles into the plasma membrane, and decreases their exocytosis in response to glucose in the circulation. It may also induce apoptotic processes in pancreatic cells, resulting in beta cell death. Figure 1 shows the potential molecular-level mechanisms with implications for the oxidative stress-beta-cell dysfunction interaction, which has as an endpoint the development of both types of diabetes [22,23,24,25].

Molecular pathways like stress-activated protein kinase/c-Jun amino-terminal kinase (SAPK/JNK), nuclear factor kappa B (NF-κB), hexosamine pathways, and p38 MAPK (mitogen-activated protein kinase) were shown to be triggered by oxidative stress in 2017 by Wang and Wang. The malfunctioning of beta cells is significantly influenced by these stress-activated signaling routes [26].

An impaired INS signal transduction (IST) is caused by INS receptor substrate (IRS)-1 and IRS-2 serine phosphorylation, which is brought on by oxidative stress [27].

By activating the SAPK/JNK signaling channels, free radicals can determine the IRS-1 phosphorylation at position 307 and decrease the normal IST. Additionally, they have the ability to suppress normal IST through p38 MAPK-dependent molecular pathways, and the blockage of this molecular pathway restored normal IST in vitro and in an animal model of DM [28].

Due to its involvement in critical processes including inflammation, glucose balance, and cell survival, oxidative stress and various diseases have a significant connection. Through a number of metabolic mechanisms, such as an increase in glycation and diacylglycerol, glucose self-activation, and the activation of protein kinase C and polyol pathways, hyperglycemia increases oxidative stress in both types of DM. Additionally, this mechanism contributes to the development and difficulties of DM because it increases lipid peroxidation by generating more free radicals and decreasing the antioxidant enzymes [29].

EOs are fluid mixes of volatile chemicals that are frequently derived from aromatic plants by steam distillation. Moreover, the methods of production of EOs encompasses various methodologies, including supercritical fluid extraction, gas-assisted mechanical expression, ultrasound-assisted extraction, and microwave-assisted extraction [30]. The biological benefits of EOs, including their antiviral, antibacterial, anti-inflammatory, and antioxidant activities, have been documented [31].

It has been demonstrated that the chemical composition of EOs is considerably influenced by environmental conditions and plant age, which changes their antioxidant properties. Since the antioxidant properties of various EOs must be compared, it is important to study their chemical profiles. Phytochemicals often serve as antioxidants through a number of mechanisms, including chelating redox-active metals, delaying the generation of free radicals, and/or scavenging them to improve endogenous antioxidant defense and lower autoxidation [32].

The treatment and control of blood glucose levels in T2DM and borderline patients can benefit from the suppression of α-amylase along with α-glucosidase, enzymes responsible for the digestion of carbohydrates. This may considerably decrease the post-prandial rise in blood glucose. Physiological effects that can be modulated by plant-based medications and functional meals in the treatment and prevention of obesity and DM are currently receiving new attention. It is possible to find many natural oral hypoglycemic medications with minimal or no adverse effects in the botanical world [33].

### Antioxidant Effects of Plants in Diabetes Mellitus

By giving free radicals reducing equivalents in the form of hydrogen atoms or electrons, several antioxidants found in the human body, like glutathione (GSH) and thioredoxin, sweep up reactive oxygen species (ROS) and decrease their toxicity to the body’s systems. According to studies on T2DM treatment, certain plant-derived substances exhibit the following properties: reduce the inflow of inflammatory cells; decrease cyclooxygenase-2-related gene expression, hence facilitating a rise in the release of proinflammatory mediators; suppress the production of the intercellular adhesion molecule and the macrophage chemo static protein; reduce the serum concentrations of the pro-inflammatory cytokines interleukin-6, interleukin-1*β*, and tumor necrosis factor (TNF-α); initiate the extracellular signal-regulated kinase 1/2 and AMPK pathway activation; suppress the NF-*κ*B pathway activity; and enhance INS sensitivity and glucose tolerance [34,35].

Both T1DM and T2DM cause a major alteration in the metabolism and structure of lipids. Hyperlipidemia is linked to lipid peroxidation. In addition to having a significant impact on DM, the liver is essential for maintaining lipid and glucose homeostasis. The synthesis of phospholipids, cholesterol, and triglycerides is carried out in the liver and kidneys, which also take part in the oxidation, absorption, and metabolism of free fatty acids. T2DM can often be successfully treated using medicinal plants even if there are anti-diabetic medications available on the market [36].

A significant number of studies have shown how using medicinal herbs with hypoglycemic characteristics can help manage T2DM. Flavonoids, phenolics, alkaloids, and tannins are the most frequently used herbal active components in the treatment of T2DM [37].

Despite the availability of numerous traditional medications such as dipeptidyl peptidase 4 inhibitors, metformin, glucagon-like peptide 1, sodium-glucose cotransporter-2, sulfonylureas, and thiazolidinediones side effects still occur. Plant extracts have been suggested to be an excellent alternative treatment for T2DM, having fewer side effects and having been documented to have several antidiabetic benefits. In an investigation, 65 recent trials on plant extracts that reduce T2DM were examined. The protein kinase B (Akt)/phosphoinositide 3-kinase (PI3K) pathway was used by plant extracts to regulate blood sugar levels. NF-κB and JNK pathways, which cause INS resistance, have been inhibited by the antioxidant and anti-inflammatory properties of plant extracts. Stimulating AMPK controls fatty acid oxidation and lipogenesis, two processes that are also linked to INS resistance [38].

Vitamin A (carotenoids), vitamin E (in its most active form, α-tocopherol), GSH, vitamin C (L-ascorbic acid), in addition to polyunsaturated fatty acids or exogenous flavonoids (rutin, resveratrol, quercetin, etc.), that could improve the body’s antioxidant system, are the essential molecules [39].

These medicines, for instance, can prevent cancer and diabetic problems by raising the level of GSH. For instance, boosting GSH levels with these products helps prevent DM and cancer [40].

Various ROS-producing enzymes, including cyclooxygenase, GSH-S-transferase, lipoxygenase, mitochondrial succinoxidase, microsomal monooxygenase, nicotinamide adenine dinucleotide oxidase, and xanthine oxidase, as well as chelating trace metals and suppressing phospholipases A2 and C, were demonstrated to be inhibited by these substances. They operate by offering a hydrogen electron or atom to the superoxide anion as well as to the alkoxyl, hydroxyl, and peroxyl radicals, shielding DNA, proteins, and lipoproteins from oxidative damage [41].

**Figure 2:**
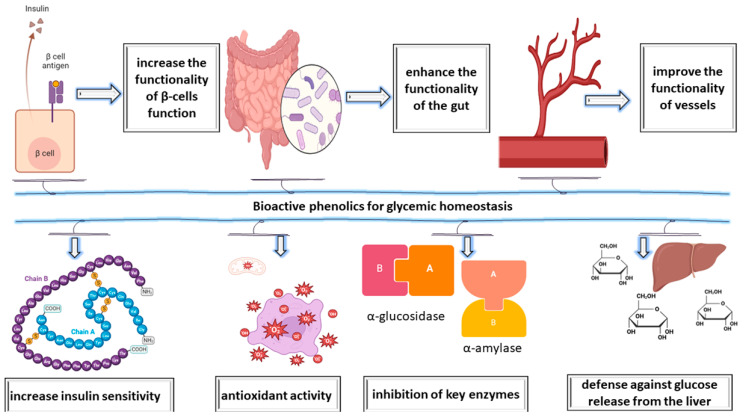
Unveiling glycemic control benefits and the functional properties of plant phenolics.

In conjunction with their antioxidant properties, the bioactive phenolic compounds (i.e., p-coumaric acid, caffeic acid, ferulic acid, benzoic acid, protocatechuic acid, and gallic acid) of various plants possess beneficial effects, including enhancing INS sensitivity, decreasing the production of glucose by the liver, hindering the activity of essential carbohydrate digestive enzymes, and regulating glucose absorption in the circulatory system, thus enhancing post-prandial glycemic management (Figure 2). Furthermore, these effective therapeutic attributes have immediate implications as well as advantages for the management of diabetes [42].

According to the literature, 410 medicinal plants have had their anti-diabetic effects experimentally established; however, only 109 of these plants have had the full mechanism of action investigated. The modulation of metabolic pathways such gluconeogenesis, glycolysis, glycogen synthesis and degradation, Krebs cycle, carbohydrate metabolism and absorption, cholesterol synthesis, and INS synthesis and release, has been demonstrated for a number of medicinal plant extracts [43].

Additionally, ROS overproduction is linked to INS resistance, and the PI3K/AKT pathway is upregulated by polyphenols, upregulating endogenous antioxidants through nuclear respiratory factor2-dependent pathways, which is advantageous for reducing INS resistance [44].

## 3. Essential Oil Types Used in Diabetes Mellitus

### 3.1. Clove Essential Oil

*Syzygium aromaticum* L. is a member of the Myrtaceae family, which includes 130–150 genera and about 3000 species of plants in the clove, eucalyptus, guava, and myrtle families. Clove is grown in China, Indonesia, Madagascar, and Sri Lanka as an aromatic flower [45].

Eugenol is the main compound in clove essential oil (CEO), making up at least 50% of the more than thirty known compounds. Eugenyl acetate, β-caryophyllene, and α-humulene account for the remaining 10–40%, less than 10% of the total belonging to minor or trace elements (including cadinene, caryophyllene oxide, chavicol, diethyl phthalate, 4-(2-propenyl)-phenol, α-cubebene, and α-copaene, and several others) [46].

An investigation analyzed how the intraperitoneal injection of CEO: 20 mg/kg body weight influenced the expression of proinflammatory mediators, specific oxidative stress enzymes, and the enzymes involved in glucose metabolism. When compared to the streptozotocin (STZ) group, the diabetic rats that received CEO had significantly lower blood total cholesterol, glucose, and xanthine oxidase levels. The concentrations of lipid peroxides and thiol groups in the brain and liver tissues of the treated rats also showed a considerable reduction. Following CEO treatment, the activity of metabolic and antioxidant enzymes returned to normal in diabetics. CEO is a powerful α-amylase inhibitor in addition to its preventive actions against red blood cell hemolysis [47].

Through the use of specific biochemical targets related to DM and, in particular, its inhibitory influence on the polyol pathway, another study aimed to assess the antioxidant and anti-diabetic effects of CEO and to establish its mechanism of action. In the previously mentioned research, the antioxidative activity and inhibitory effects of CEO on aldose reductase were investigated in vitro, in vivo, and in silico. According to in silico docking experiments, all the CEO compounds that were chosen had an energy change that ranged between −5.5 and −8.8 kcal/mol and a constant of inhibition that ranged between 357.08 nM and 93.12 µM. Non-competitively, CEO strongly inhibits aldose reductase. In Sprague Dawley rats that had STZ-induced DM, CEO supplementation at 20 mg/kg BW reduced retinal sorbitol dehydrogenase function through decreased aldose reductase activity. Furthermore, CEO injections in diabetic rats contributed to improved glycemia levels [48].

### 3.2. Peppermint Essential Oil

Mentha, a genus of plants of the Lamiaceae (mint family) botanical family, that is also known as peppermint, is extensively distributed across regions with temperate climates. There are many different of compounds in menthe that are both essential and non-essential, including peppermint EO (PEO). PEO is a mixture of volatile metabolites with antioxidant, anti-inflammatory, antiviral, antibacterial, scolicidal, anti-tumor, immunomodulatory properties, having menthol, neomenthol, menthone, and iso-menthone as its major constituents. Increasing data suggest that PEO could exhibit hypolipidemic and hypoglycemic effects, as well as provide pharmacological protection for the brain, skin, liver, gastrointestinal, kidney, respiratory, and neurological systems [49].

The goal of this experimental investigation was to determine whether PEO has any capacity to reduce blood sugar levels in rats with diabetes induced with nicotinamide and STZ. A single dose of STZ and an intraperitoneal injection of nicotinamide were used to cause DM in rats that had been fasting the previous night. After 72 h, one group of diabetic rats received the common hypoglycemic drug glibenclamide, while the other two groups of rats with DM received PEO at different concentrations (40 and 80 mg/kg BW). Estimates were made for the concentrations of C-peptide, serum INS, and blood glucose. Quantitative analysis was performed for the oxidative stress indicators; as for histological analysis, pancreas and liver samples were gathered. For evaluating the expression of INS and B-cell lymphoma 2 (Bcl-2) in the pancreas and liver, immunohistochemical assays were conducted. Following PEO therapy, it was identified that the anemia caused by DM was corrected, the concentrations of blood glucose were decreased, and the levels of serum C-peptide and INS were elevated. Leukocyte and platelet counts, which became lower during DM, had also increased [50].

### 3.3. Lavender Essential Oil

Lavender, as commonly known in English, comprises anthocyanins, coumaric acid, coumarins, EOs, glycolic acid, herniarins, minerals, phytosterols, sugars, tannins, ursolic acid, and valeric acid. It was traditionally utilized for the treatment of wounds, biliousness, dreadful migraines, colic, and chest disorders. Besides those, it possesses antibacterial, anti-diabetic, antifungal, antimicrobial, anti-parasitic, analgesic, and neurologic properties [51].

The EOs of *Lavandula stoechas* (French lavender) obtained in the region of Ain-Draham (northwest Tunisia) were presented in a study considering their phytochemical profile in addition to their protective properties against oxidative stress and DM in rats with alloxan-induced DM. By hydro distilling plant aerial parts, samples of EOs were obtained. The samples were then evaluated by gas chromatography–mass spectrometry (GC-MS). Four groups of rats were formed: healthy control, diabetic control, healthy with EOs, and diabetic with EOs.

After the subacute intraperitoneal administration of *Lavandula stoechas* EOs (50 mg/kg i.p., b.w.) to rats for 15 days, the antioxidant and anti-diabetic effects were assessed. Endobornyl acetate (1.03%), limonene (2.71%), linalool (2.01%), eucapur (3.29%), α-pinene (23.18%), camphor (15.97%), D-fenchone (29.28%), and camphene (7.83%), were the main compounds found. Smaller amounts of cymene, tricyclene, selina-3,7(11)-diene, and delta-cadinene were also present in the EOs. Additionally, it was shown that *Lavandula stoechas* EOs provided a strong defense against both the decline in antioxidant enzyme activity and the rise in blood sugar brought on by aloxan administration. The subacute treatment with EOs resulted in an increase in the activity of antioxidant enzymes and a reduction in lipoperoxidation [52].

### 3.4. Origanum Essential Oil

Oregano EOs are well known for their antibacterial characteristics, in addition to being antiviral and antifungal. Furthermore, current research has shown that these substances are also effective cancer suppressors, anti-inflammatory, antioxidant, and anti-diabetic agents. The cosmetic, food, and pharmaceutical industries may find these oregano EO qualities useful. Reviewing the most recent research on oregano EOs and their health benefits is one of the objectives of this research [15].

The antimicrobial, antioxidant, and inhibitory properties of EOs from *Origanum vulgare* subsp. hirtum (OVH) and *Origanum vulgare* subsp. vulgare (OVV) were assessed. These activities included 2,2′-azinobis-3-ethylbenzothiazoline-6-sulfonic acid (ABTS), CUPric Reducing Antioxidant Capacity, 2,2-diphenyl-1-picrylhydrazyl (DPPH), ferric reducing ability of plasma (FRAP), phosphor-molybdenum β-carotene/linoleic acid, and metal chelating. The major components of the OVV and OVH Eos were determined to be thymol and linalool. OVV demonstrated potent antibacterial, butyl cholinesterase, acetylcholinesterase, α-glucosidase, and α-amylase inhibitory, reducing the activity and free radical scavenging properties [53].

### 3.5. Oliveria decumbens, Thymus kotschyanus, Trachyspermum ammi, and Zataria multiflora Essential Oils

The incorporation of various EOs into a composite made of gelatin and pectin, including *Zataria multiflora*, *Thymus kotschyanus*, *Oliveria decumbens,* and *Trachyspermum ammi*, was studied. According to the GC-MS analysis, the main components of the EOs include carvacrol (3.2–52.4%), geraniol (0.0–14.5%), gamma-terpinene (0.0–12.7%), paracymene (3.2–5.2%), spathulenol (0.0–13.6%), and thymol (1.2–86.4%). The gelatin–pectin composite that contained EOs had a negative zeta potential of 14.2–16.9 mV, a low conductivity of 265–278 µS/cm, a low surface tension of 19.0–23.5 mN/m, a low Newtonian viscosity of 23.7–28.5 mPa.s, an acidic pH of 2.40–3.04, and a nanoscale particle size of 313–336 nm. Due to these rheological characteristics, globular gelatin–pectin nanoparticles having sizes between 500 and 700 nm were formed. The gelatin–pectin and gelatin–pectin-EOs FTIR spectra were quite similar, pointing to their noncovalent interactions. The EO-infused gelatin–pectin composite demonstrated antiamylase activity (216–230 µg/mL), antiglucosidase activity (212–238 µg/mL), antiprotein oxidation (150–168 µg/mL), antiglucose oxidation (130–150 µg/mL), antiprotein glycation (145–170 µg/mL), and antilipid peroxidation (120–130 µg/mL) [9].

### 3.6. Nigella Sativa Essential Oil

The Ranunculaceae family includes *Nigella sativa* L. (NS), an annual herbaceous flowering plant that is mainly prevalent in the Middle East. It has long been utilized for culinary purposes as a condiment or a spice and is commonly referred to as black cumin, black seed, or kalonji. Traditional medicine has used various NS dosages and forms including extract, powder, and oil to treat a range of conditions, including gastrointestinal disorders, diarrhea, bronchitis, cough, and fever [54].

The potential use of black cumin for the control of DM has recently attracted the attention of researchers from all over the world. Blood glucose levels were reduced by NS extracts, commonly referred to as black cumin or black seeds; however, the precise anti-diabetic mechanism is still unknown. Black cumin oil hypoglycemic properties may be attributed to the presence of certain phytochemicals, such as carvacrol and thymoquinone [55].

Examining the antidiabetic potential of *Nigella sativa* fixed oil (NSFO) and *Nigella sativa* EO (NSEO) was a key objective of another study. Throughout the whole study period, three experimental groups of rats—D1 (control), D2 (4.0% NSFO), and D3 (0.30% NSEO)—received dietary regimens. The lipid profile was modified by the experimental diets (NSEO and NSFO), and the antioxidant damage was reduced. However, in the control group, the generation of free radicals, specifically MDA, increased by 59.00%, and conjugated dienes increased by 33.63%. The levels of MDA were decreased by 11.54 and 26.86% and the levels of conjugated dienes by 32.53 and 38.39%, respectively, with NSFO and NSEO. The health benefits of N. sativa oils included several favorable anti-diabetic effects [56].

Additionally, it has been suggested that NS is a safer alternative to oral medications for DM [57]. Due to its potential medicinal benefits, researchers are now more interested in searching for novel bioactive substances from NS. Thymoquinone, one of the key bioactive molecules that has been shown to possess protective properties against DM, is primarily responsible for the therapeutic actions of NS [58].

Previous research found that thymoquinone significantly reduced fasting blood glucose (FBG) and increased INS levels in rats [59].

A total of 46 diabetic undergoing hemodialysis patients enrolled in a randomized, double-blind, and placebo-controlled clinical investigation. Participants were randomly assigned to the NS (n = 23) or placebo (n = 23) groups. For 12 weeks, paraffin oil was given to the placebo group while to the NS group was administered 2 g/day of NS oil (NSO). The following serum values were assessed before and after the study: FBG, glycosylated hemoglobin (HbA1C), high-sensitivity C-reactive protein (hs-CRP), INS, malondialdehyde (MDA), superoxide dismutase (SOD), and total antioxidant capacity (TAC). TAC, SOD, and INS levels rose in comparison to baseline values, while HbA1c, hs-CRP, FBG, and MDA levels considerably reduced. The analysis of covariance (ANCOVA) test was used following the covariates adjustment, showing that changes in the MDA, FBG, HbA1c, TAC, hs-CRP, and SOD, levels were statistically significant when compared to the placebo group. The INS level did not show any substantial intergroup variations. Furthermore, no significant side effects were observed throughout the study [60].

For evaluating the impact on the lipid profile, blood glucose, gene expression of various INS receptor-induced signaling molecules, and oxidative stress variables, rats with STZ-induced diabetes were fed a high-fat diet received daily treatment with NSO. The INS receptor inhibitor I-OMe-AG538 and a few other medications (glimepiride and metformin) were also administered in this treatment regimen. Comparing rats who received NSO to those who did not, the NSO treatment considerably increased the gene expression of the INS receptor. Additionally, it downregulated the expression of the TNF-α converting enzyme (TACE; also known as adisintegrin and metalloprotease 17; ADAM17) while upregulating the expression of PI3K and INS-like growth factor-1. The examination of the tissue inhibitor of the metalloproteinases-3 content confirms the expression of ADAM-17. Additionally, the NSO dramatically decreased the serum INS/INS receptor ratio, lipid profile parameters, TNF-α, oxidative stress indicators, and blood glucose levels. These results support the NSO antidiabetic effect [61].

Another investigation looked at how NSO influenced the levels of FBG, oxidative stress, systemic inflammation, and lipid profile in type 2 diabetic mellitus (T2DM) patients. A total of 50 T2DM patients were included in a double-blind and randomized clinical trial investigation. Participants were randomly assigned to placebo or NSO groups. In contrast to the placebo group, which received a similar placebo, the treatment group was administered 1000 mg of NSO in two capsules twice a day for eight weeks. For measuring the serum MDA, lipid profile, hs-CRP, and FBG, following a 14 h fast, 5 mL of blood from each patient was analyzed at the beginning and end of the investigation. In order to compare the examined parameters for the two groups while monitoring for specific variables, covariance analysis was conducted. When compared to the placebo group, the administration of NS supplement to the intervention group was substantially correlated to reductions in total cholesterol, HDL cholesterol, triglycerides, MDA, FBG, and serum hs-CRP, and a raise in the serum levels of LDL cholesterol [62].

El-Dakhakhny et al. pointed out that the hypoglycemic impact of Nigella sativa (NS) is not directly correlated with the activation of INS secretion. In their investigation, they observed that rat pancreatic islets may be stimulated to secrete INS by NSO and its bioactive components, thymoquinone and nigellone, when glucose was present. The researchers hypothesized that the hypoglycemic effect associated with NS may be attributed to its extra pancreatic function, as the release of INS does not correspond to the blood glucose level. However, the researchers employed a significantly elevated dosage of STZ (75 mg/kg) to induce DM in the experimental animals, resulting in more profound impairment to the β cell. This approach closely resembles the pathophysiological characteristics of T1DM rather than T2DM, as documented in the earlier investigation [63].

### 3.7. Salvia Essential Oil

The term Salvia originates from the Latin term *salvare*, which carries the connotations of healing and preservation. Salvia species have been found to synthesize several phenolic compounds, and these substances have received significant interest due to their potential cytotoxic, antibacterial, antioxidant, neuroprotective, anti-inflammatory, and antidiabetic activities [64].

*Salvia officinalis* has been used since ancient times in ethnomedicine for the treatment of various ailments such as inflammation, rheumatism, gout, tremor, disorientation, seizures, paralysis, hyperglycemia, ulcers, and diarrhea. In recent years, numerous studies have been conducted to demonstrate the effects for which it was traditionally used and explore its potential novel biological properties. The experiments conducted have demonstrated a diverse array of pharmacological effects, encompassing antioxidant, anticancer, anti-inflammatory, antibacterial, anti-dementia antimutagenic, hypoglycemic, hypolipidemic, and antinociceptive effects [65].

Previous research has indicated that the administration of sage extracts has the potential to improve glycemic regulation in both healthy and diabetic animal models. A water ethanolic extract of S. officinalis administered intraperitoneally, which produced a decrease in glucose levels in fasting normoglycemic mice as well as in mice that had mild DM caused by alloxan, as demonstrated by Alarcon-Aguilar et al. in 2002 [66]. Furthermore, it has been demonstrated in an experimental study that the intraperitoneal administration of sage methanolic extract resulted in a considerable reduction in serum glucose levels in fasting rats with STZ-induced diabetes. Notably, this reduction occurred without any modifications in plasma INS levels [67]. In another study, when diabetic rats receiving STZ were compared to normal diabetic rats, the ethanolic extract of sage dramatically reduced the serum total cholesterol triglycerides and glucose, while increasing the serum INS levels [68].

The assessment of the antidiabetic impact in vitro involved the examination of the inhibitory effects on the activities of α-glucosidase, α-amylase, and lipase. Additionally, the anti-inflammatory effect was assessed by the utilization of the 5-lipoxygenase test. Agar well diffusion assay and the microdilution method were also used to evaluate the antibacterial efficacy against six bacterial strains. Naphthalenone, α-thujone, 1.8-cineole, and camphor were the predominant components in the EOs of S. officinalis during the three different phenological phases. EOs extracted from the plants at their peak of flowering demonstrated the highest levels of antioxidant activity across all tests. At the full flowering stage, this oil demonstrated significant inhibitory effects on α-glucosidase, α-amylase, and lipase enzymes. The inhibiting impact of 5-lipoxygenase was found to be most pronounced during the full flowering stage [69].

*Salvia officinalis* L. (Sage) EO was tested for the first time in a study examining its impact on Alloxan-triggered DM in male Wistar rats. Gas chromatography coupled with flame ionization detection (GC-FID) and gas chromatography coupled with mass spectrometry (GC-MS) were used to examine the EO of sage after it had been extracted using a Clevenger device. Hydrocarbon monoterpenes (15.00%), oxygenated monoterpenes (56.32%), and hydrocarbon sesquiterpenes (14.70%) were the three chemical families that were found to be the most abundant in this oil. The administration of all treatments in the mentioned study was conducted orally. Research conducted in vitro revealed that the EO exhibited inhibitory effects on α-amylase and lipase enzymes. Serum lipase and α-amylase activity were found to be lowered by 32.1% and 46.6% in in vivo investigations. Both blood sugar and liver glycogen were lowered by sage EO, by 60 and 43%, respectively. Sage EO has been shown to considerably protect liver function by reducing serum alanine aminotransferase (ALT) (79%), lactate dehydrogenase (LDH) (43%), and alanine aminotransferase (AST) (35%). Moreover, the administration of Sage EO has shown efficacy in maintaining renal function, as shown by the significant reduction in serum creatinine levels by 47% and uric acid concentrations by 62.5%, resulting in values comparable to those observed in the control group [70].

### 3.8. Citrus Aurantifolia Essential Oil

The genus Citrus, belonging to the plant family Rutaceae, is generally recognized to be part of the most frequently consumed and geographically widespread fruit varieties across the globe. There are various types of citrus fruits, such as lemons, limes, mandarins, oranges, pomelos, tangerines, and hybrids. Citrus fruits hold significant economic value due to their high consumption rates [71].

The nutritious benefits and delicious flavors of these fruits make them a popular choice for immediate consumption in the forms of whole fruits or juices [72]. Polyphenols and carotenoids are widely recognized for their multiple health advantages, mostly accredited to their potent antioxidant properties. The manufacturing of pharmaceuticals, cosmetics, and functional foods could greatly benefit from the use of polyphenols as a profitable raw material [73].

EOs from citrus fruits are complex combinations of oxygenated chemicals, terpene hydrocarbons, and non-volatile residues such as esters, alcohols, aldehydes, terpenes, and sesquiterpenes. When it comes to distinguishing between citrus kinds, the volatile chemical substances found in the EOs serve as the most useful indicators. Significant roles in antidepressant, anti-inflammatory, antioxidant, antifungal, and anticarcinogenic activities are played by the active components of citrus EOs, including α-terpineol, β-pinene, γ-terpinene, d-limonene, geranyl acetate, linalool, linalyl acetate, and terpinolene [74,75].

The antidiabetic potential of an EO from the leaves of the citrus aurantifolia tree was evaluated in alloxan-induced hyperglycemic rats with metformin serving as the standard of comparison. The oil was extracted using hydro distillation. Based on the chemical examination, the main component of the oil was found to be D-limonene (57.84%). Neral (7.18%), linalool (4.75%), sulcatone (3.48%), and isogeraniol (3.48%) were also shown to be significant chemicals. Hyperglycemic rats treated with C. aurantifolia oil (100 mg/Kg b.wt. intraperitoneally daily for 14 days) showed a significant decrease in FBG and hepatic glucose and an increase in the hepatic glycogen concentration [76].

### 3.9. Black Pepper Essential Oil

Black pepper (Piper guineense) seed EO was tested for its antioxidant capabilities and its influence on α-glucosidase, α-amylase, and angiotensin-I converting enzyme (ACE), three critical enzymes connected to both T2DM and hypertension. The EO exhibited a scavenging activity against DPPH∗, NO∗, and ABTS∗ radicals, as well as demonstrated a chelating ability towards Fe^2+^ ions. GC analysis revealed a variety of compounds, including 1,8-cineole, cis-ocimene, myrcene, allo-ocimene, and α—and β -pinene. The concentration-dependent inhibition of α-glucosidase, α-amylase, and ACE enzyme activity was observed with the EO; α -glucosidase inhibition was more pronounced than α-amylase inhibition. To summarize, the EO of black pepper may have potential in preventing and/or managing hypertension and T2DM, due in part to its phenolic content, the inhibition of α-glucosidase, α-amylase, and ACE activities and antioxidant activity [77].

### 3.10. Rosemary Essential Oil

*Rosmarinus officinalis* L., commonly referred to as rosemary, is a botanical species included in the family Lamiaceae and is native to the Mediterranean region. It can also be observed in various regions across the globe. The plant in question is perennial and fragrant, characterized by its shrub-like form and abundant foliage. It typically reaches a maximum height of two meters and possesses green leaves that emit a distinctive scent. In addition to its culinary and therapeutic uses, *R. officinalis* is also a beautiful decorative plant [78].

The extracts and EOs of *Rosmarinus officinalis* L. (ROEO) contain a wide range of chemicals, some of which have been shown to have pharmacological effects, but their concentrations vary from plant to plant. Alpha-pinene, caffeic acid, camphor, chlorogenic acid, carnosol, derivatives of eugenol and luteolin, eucalyptol, monomeric acid, oleanolic acid, rosmanol, rosmarinic acid, rosmadial, rosmaquinones A and B, ursolic acid, and secohinokio are among the most commonly reported phytocompounds [79].

A study set out to find out whether ROEO could prevent DM and oxidative stress in rats that had been given alloxan. There were four distinct groups of animals under study: the controls—HC, the diabetics—DC, the healthy with ROEO (H + ROEO), and the diabetics with ROEO (D + ROEO). Researchers identified 15 different chemicals in ROEO using GC/MS. Hyperglycemia, dysfunctions of the liver and kidneys, and the dysregulation of lipid metabolic markers were reported to be produced by alloxan administration. In both liver and kidney tissues, alloxan administration resulted in an oxidative stress status as measured by a decrease in thiol groups (-SH) levels, an increase in MDA content, and a depletion of antioxidant enzyme activities such as the total SOD, catalase (CAT), Fe-SOD, Cu/Zn-SOD, and Mn-SOD. The subacute treatment with ROEO for 15 days has considerably reversed all metabolic abnormalities brought on by alloxan intoxication [80].

Chronic renal failure and other kidney issues are the last result of diabetic nephropathy, an extremely serious disease. Rosemary has several medicinal uses, including its ability to fight cancer, DM, inflammation, blood clots, and liver damage. The effects of ROEO, INS, and a combination of the two were compared regarding their effects on histological, immunohistochemical, and biochemical kidney changes in STZ-induced diabetic rats. Thirty-six adult albino rats were randomly assigned into several groups: normal control that were non-diabetic; diabetic (intraperitoneal STZ, 55 mg/kg); diabetic ROEO-treated (ROEO, 10 mL, nasogastric gavage); diabetic INS-treated (Lantus INS, 2 units/day, SC); and diabetic INS and ROEO-treated. Histological, immunohistochemical, and biochemical examinations were carried out. When compared to the control group, those with DM showed significantly higher levels of urea, uric acid, glucose, and creatinine; an increase in glomerulosclerosis; an elevated score of tubular injury; increased levels of MDA and catalase (CAT) in kidney homogenates; and a significant reduction in overall GSH as well as in SOD [81].

### 3.11. Periscaria hydropiper Essential Oil

Medicinal plants and EOs have gained recognition for their wide range of biological actions, which encompass the ability to alleviate DM. The purpose of the study was to analyze the phytochemical composition and investigate the in vitro anti-diabetic properties of EOs isolated from the leaves of *Persicaria hydropiper* L. (*P. hydropiper*). Using a hydro distillation device, the EOs from P. hydropiper leaves (Ph.Los) were extracted. These oils were subsequently processed in a phytochemical investigation using the GC-MS method. Ph.Lo was evaluated against α -glucosidase and α-amylase, two crucial enzymes that are significant targets in T2DM. The binding affinities of the determined substances against the enzyme targets were evaluated in silico using the MOE-Dock program. Acarbose, the positive control, showed an inhibitory activity of 77.30 ± 0.61% percent at the same dose [82].

### 3.12. Momordica charantia Essential Oil

The climbing annual plant *Momordica charantia* L. (MC), often called bitter melon, bitter gourd, or karela, belongs to the Cucurbitaceae family. Although it was originally from East India, currently, it is cultivated and consumed all around the world, including in colder climates. The vegetable has a long, narrow cone shape, and a pale green color. Despite its bitter flavor, it is widely consumed due to its numerous health advantages [83]. This plant is a nutrient powerhouse. Ascorbic acid, flavonoids, minerals, polysaccharides, proteins, saponins, steroids, triterpenes, and vitamins, are just some of the many phytochemicals found in MC as research into the plant continues to progress [84].

Researchers have isolated an INS receptor (IR)-binding protein (mcIRBP) in MC. These were the bioactive peptides from the protease-digested mcIRBP that were discovered to be gastro-resistant and hypoglycemic. Through the utilization of in vitro digestion and an IR kinase activity test, it was determined that mcIRBP-9, a peptide consisting of nine amino acid residues, exhibited gastro-resistance properties and effectively augmented IR kinase activities. The activation of mcIRBP-9 induced the transduction cascade of INS receptor (IR) signaling, leading to the phosphorylation of IR, the repositioning of glucose transporter 4 (GLUT4), and subsequent glucose uptake within cells. STZ-induced diabetic mice had their glucose clearance accelerated by 30.91 ± 0.39% after the intraperitoneal treatment with mcIRBP-9 and by 32.09 ± 0.38%, after oral administration [85].

In research, the focus was on a protein called mcIRBP, which may have interactions with IR. Both the structural and functional connections involving mcIRBP and IR have been examined in detail. By using photo-cross-linking and mass spectrometry, it was determined that the three distinct sections of mcIRBP physically interacted with the IR leucine-rich repeat domain and the IR cysteine-rich area. INS and mcIRBP revealed collaborative binding actions in an IR-binding experiment. When bound to IR, mcIRBP promoted a glucose uptake in 3T3-L1 cells by activating IR’s kinase activity, elevating the phospho-IR protein levels, and influencing the PI3K/Akt pathways [86].

Another attempt to investigate the potential anti-diabetic properties of MC focused on isolating EO using the seeds of the plant. Hydro distillation was used to extract the oil and GC-MS was used to examine the phytochemicals. Additionally, acute toxicity studies were conducted on rats. Serum lipid profile, protein, blood glucose, liver glycogen, and other serum indicators including AST, ALT, ALP, creatinine, and urea were measured in EO-treated STZ-induced diabetic rats to assess its anti-diabetic effects. Histological alterations in the liver, kidney, and pancreas were assessed through the use of eosin and hematoxylin staining. Using GC-MS, the hypoglycemic and INS-inducing properties of the phytochemicals were determined. Treatment with EOs significantly decreased the blood glucose levels. EO administration repaired disturbances in the metabolism affecting diabetic control rats, including those in enzymes, creatinine, glycogen, lipid profile, protein, and urea. Additionally, treated rats showed a reversal in the histologic alterations in their essential organs [87].

### 3.13. Blepharispermum hirtum Essential Oil

A study was conducted to investigate the composition of the EO extracted from the stem and leaves of *Blepharispermum hirtum* Oliver (Asteraceae) using hydro-distillation and analyzed using the GC-MS technique, marking the first example of such exploration. A comparative analysis of the EOs derived from the stem and leaves of *B. hirtum* was conducted. The objective was to evaluate their potential as in vitro anticancer and antidiabetic agents. To assess the antidiabetic activity, an in vitro α-glucosidase test was employed. Additionally, an MTT inhibition assay was used to evaluate the anticancer potential of the EOs. Similar amounts of fifty-eight compounds were found in the stem and leaves EOs of *B. hirtum*, accounting for 93.88 and 89.07% of the total oil content, respectively. Camphene was found to be the most abundant chemical in the stem EO at 23.63%, while other components were: laevo-β-pinene (4.38%), β-elemene (4.66%), and β-selinene (5.33%). The predominant chemical in the EO from leaves was discovered to be 24-norursa-3,12-diene (9.08%), with caryophyllene oxide (5.62%), β-selinene (7.26%), β-eudesmol (7.81%), and thunbergol (5.84%) following closely. When *B. hirtum* was tested to acarbose, it was found that the stem had significantly more antidiabetic potential than the EO of the leaves [88].

### 3.14. Eucalyptus Essential Oil

Anti-diabetic properties were also observed in the EO from *Eucalyptus globulus* leaves. Its effectiveness in inhibiting α-amylase was approximately twice that of acarbose when tested in vitro. Antioxidant efficiency, as measured by free radical scavenging activity and inhibition of pancreatic lipase, was also detected. The primary components of the studied EO were aromadendrene (10.1%), α-pinene (13.6%), 1,8-cineole (43.2%), and 4-carene (6.9%). In this case, it was also speculated that phenolic compounds were responsible for the results achieved [89].

### 3.15. Melissa officinalis Essential Oil

*Melissa officinalis* (MO) is a perennial herbaceous plant renowned for its aromatic properties, sometimes referred to as honey balm, lemon balm, or bee balm. It is classified in the Lamiaceae family, commonly known as the mint family. Specifically, it is a member of the genus Melissa L., which includes other species such as *Melissa flava*, *Melissa axillaris*, and *Melissa yunnanensis*. The MO square or quadrangular stem can reach heights of 0.5 to 1.5 m, typical for members of the Lamiaceae family. The green ovate to cordate leaves grow in decussate pairs and are utilized for medicinal purposes due to their high concentration of active substances [90].

Numerous studies have examined the composition of MO EO, and the results suggest that it primarily consists of volatile compounds like monoterpenes and sesquiterpenes citrals (neral and geranial, that offer citrus-like aroma), citronellal, geraniol, β-caryophyllene, and thymol, though the exact ratios between these components may vary. The process of extracting the EO, the temperature and pressure during distillation, the local environment, the plant type, and the stage of ripeness all play a role in determining the presence and concentration of any additional substances in the MO [91].

Researchers looked into the hypoglycemic effect of lemon balm (*Melissa officinalis* L.) EO (MOEO) and its antioxidant activity against DPPH radicals in db/db mice. When MOEO was diluted 270 times, it still neutralized 97% of DPPH radicals. After 6 weeks of treatment with MOEO, mice showed considerably higher serum levels of INS, an increased glucose tolerance as measured by a test for oral glucose tolerance, and considerably lower blood glucose (65%) and TAG levels compared to the control group. Gene and protein expression examinations employing RT-PCR and Western blotting techniques provided a greater understanding of the hypoglycemic mechanism of MOEO. The expression of adipocyte GLUT4, hepatic glucokinase and GLUT4, peroxisome proliferator-activated receptor (PPAR)-alpha, PPAR-gamma, and sterol regulatory element-binding protein-1c were all considerably up-regulated in the livers of the LBEO group, while the expressions of phosphoenolpyruvate carboxy kinase and glucose-6-phosphatase were down-regulated [92].

For determining whether or not *Melissa officinalis* EO (MOEO) had a beneficial impact on hyperalgesia in rats with STZ-induced diabetes, the formalin test was used. There were four groups of animals: controls, controls treated with MOEO, diabetics, and diabetics treated with MOEO. Four weeks after developing hyperglycemia, adult male Wistar rats were subjected to nociceptive tests. All rats were weighed, and their plasma glucose levels were measured once the experiment was complete. Considerable hyperalgesia was observed in both stages of the formalin test in people with DM. Control rats showed less intense nociceptive responses in both phases of the test after receiving MOEO (0.02 and 0.04 mg/day), while the diabetic rats treated with MOEO (0.04 mg/day) experienced a complete reversal of hyperalgesia. Both high doses of MOEO were effective in treating DM in animal models, with the restored euglycemia and decreased body weight in comparison to untreated diabetic mice. No significant differences in pain responses were seen between the control and DM groups after the administration of MOEO (0.01 mg) [93].

### 3.16. Rhaponticum acaule Essential Oil

*Rhaponticum acaule* (L.) DC. is a medicinal herb that is frequently employed in traditional medicine for the therapeutic management of many ailments, including gastrointestinal infections. The following presented study provides an analysis of the phenolic compound content, quantity, and antioxidant activity of several components of the plant under investigation. According to the findings, the phenolic content was high in the methanolic extracts of all three components tested. The organic extracts of the leaf were found to have the greatest flavonoid concentrations. The DPPH scavenging capacity test and the FRAP test, both of which were used to evaluate the antioxidant activity of the root extract, found that the methanolic extract of the root had the maximum activity. Five phenolic acids (chlorogenic, caffeic, ferulic, syringic, and sinapic), one flavanone (naringenin), one flavonol (rutin), and vanillin were identified through the RP-HPLC-PDA analysis [94].

*Rhaponticum acaule* (L.) DC (*R. acaule*) floral EO was analyzed by GC-MS to determine its chemical composition. The EO from *R. acaule* (RaEO) was tested for its antioxidant properties utilizing phospho-molybdenum, the reducing power, and DNA nicking assays in addition to ABTS. Xanthine oxidase, α-glucosidase, and pancreatic lipase were all tested to see how effectively they could be inhibited by RaEO. To learn more about how these EO constituents exert their inhibitory effect, researchers analyzed enzyme kinetics using Michaelis–Menten and the resulting Lineweaver–Burk (LB) plots. The analysis uncovered twenty-six chemicals (97.4%). Germacrene D accounts for 49.2% of the total, whereas methyl eugenol accounts for 8.3%, (E)-β-ionone accounts for 6.2%, β-caryophyllene accounts for 5.7%, (E,E)-α-farnesene accounts for 4.2%, bicyclogermacrene accounts for 4.1%, and (Z)-α-bisabolene accounts for 3.7%. Based on the results of the kinetic inhibition investigation, EO was found to be a mixed inhibitor and a powerful inhibitor of α-glucosidase [95].

## 4. Clinical Research on the Applicability of Essential Oils in Glycemic Control and Toxicological Considerations

**Figure 3:**
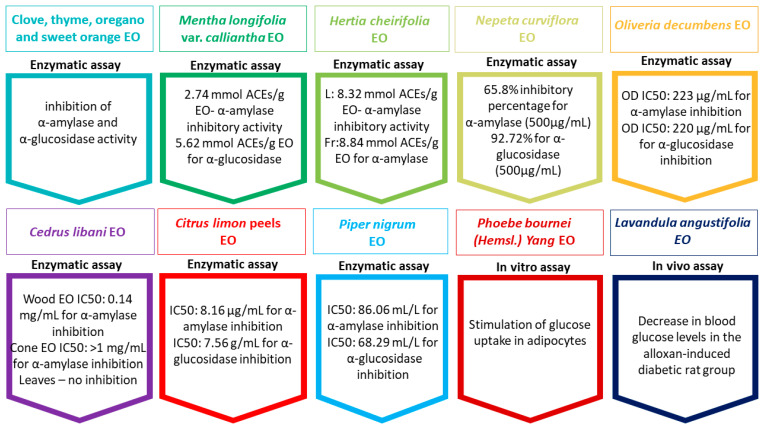
Evaluations of the effect of essential oils on diabetes prevention and treatment. Essential oil (EO); leaves (L); fruits (Fr); equivalents of acarbose (ACE); half-maximal inhibitory concentration (IC50).

The inhibition of alpha-glucosidase and alpha-amylase, reduction in gluconeogenesis, and the enhancement of sensitivity to INS or INS secretion were identified as the primary mechanisms behind the antidiabetic activity of the EOs. Moreover, Figure 3 shows a series of outcomes from enzymatic assays and in vitro and in vivo studies on the effect of EOs from certain species on inhibiting certain enzymes involved in digestion, promoting glucose uptake in adipocytes, and lowering blood glucose levels [9,52,77,96,97,98,99,100,101,102].

The clinical examination of EOs with antidiabetic potential provides crucial insights into their efficacy, safety, and overall suitability for diabetes management. However, although there are numerous experimental studies that indicate the anti-diabetic potential of EOs, few of them have been investigated in clinical studies (Table 1). Until now, only black cumin (*Nigella sativa* L.) and cumin (*Cuminum cyminum* L.) EOs have been tested on patients with diabetes in randomized clinical trials. Black cumin EO treatment, with a dose equivalent to the oil obtained from 0.7 g of seeds (approx. 0.18 mL/day), resulted in a significant decrease in (FBS) and an increase in INS levels without changing the platelet or total leukocyte counts, when compared to the control levels, and with good hepatic and renal safety in patients with T2DM [103].

The effects of cumin EO in different doses on diabetes treatment and prevention were investigated in two clinical studies that included diabetic patients [104,105] and other three studies with patients with metabolic syndrome [106], prediabetes [107], or overweight [108]. A daily dose of 50 and 100 mg of C. cyminum EO over an 8-week period resulted in considerably higher changes in the serum levels of INS, FBS, HbA1c, highly sensitive C-reactive protein (hsCRP), and TNF-α compared to a placebo in one clinical randomized investigation [104]. Another clinical study that aimed to assess the impact of cumin EO and vitamin E on the lipid profile and blood levels of leptin and HbA1C in individuals with diabetes showed that 25 mg of cumin EO per day has a greater effect and is superior to vitamin E in terms of lowering the diabetes index [105]. In contrast with these results, when it was tested on patients with metabolic syndrome or prediabetes, a dose of 75 mg/day did not bring significant improvements in the glycemic profile (fasting serum INS, FBS, and HbA1c) [106,107]. Taghizadeh et al. found that overweight subjects who consumed 100 mg/d cumin EO for 8 weeks observed improvements in the homeostatic model assessment of INS resistance (HOMA-IR) and INS, but no significant changes in FBS [108].

The heterogeneity and discrepancies among the research findings can be explained by the differences in a few variables, including different dosages, approaches, participant selection processes, length of the study, study adherence, and concentrations of bioactive compounds. Thus, further large-scale clinical trials are required to determine the therapeutic benefit of EOs that have the potential to be antidiabetic.

EOs having antidiabetic potential have been studied for their promising impact on health, but their toxicological features and primary chemical ingredients have only been studied in a small number of studies. When compared to the mostly hydrophilic extracts utilized in ethnomedicine, the toxicity profile of the EOs is very different from those of the herb itself since EOs are volatile and lipophilic, successfully crossing membranes. High levels of monoterpenes (molecules with a low molecular weight and a high lipophilicity) give these plants their distinctive characteristics, making it possible for them to cross biological barriers and thus they may harm more than one organ [109]. Monoterpenes can be found in abundance in many plant categories. While the EOs are generally considered safe for human use, their excessive use can be dangerous.

According to a recent review [110] that characterized the toxic nature of several monoterpenes (camphor, α -terpinene, thujone, limonene, citral, and pulegone), in vitro and in vivo, the most toxic are camphor, thujone, and pulegone. These can cause damage to the nervous system, DNA, or the developing fetus [110]. In human studies, thujone and 1,8-cineole have been linked to epileptiform convulsions, particularly in infants with a family history of epileptic disorders [111]. Other investigations both in vitro and in vivo have revealed the toxicity of EOs high in monoterpenes [111,112,113]. Following the sub chronic treatment with peppermint EO at a dosage of 100 mg/kg/day, the occurrence of carcinogenic effects, specifically hepatocellular carcinoma at greater concentrations, as well as nephropathy characterized by the production of hyaline droplets, was seen [112]. The peppermint EO has the capacity to interact with human lymphocytes and elicit chromosomal aberrations [113]. It must be guaranteed that the combined quantity of menthofuran and pulegone in the prescribed daily dosage remains below 37.5 mf for adult individuals. In order to reach the maximum thresholds, it is necessary to utilize peppermint oil of a sufficient grade, as determined by the specified limits of menthofuran and pulegone [112,113].

For black cumin and cumin EOs, no severe adverse effects were reported in animal or human studies [114,115,116]. Cumin EO has been shown to have side effects, such as dermatitis and respiratory problems in rare cases, when used in human studies [115]. In animal studies, at doses <500 milligrams per kilogram per day, no noticeable clinical signs or side effects were seen [116].

As the existing data are insufficient and occasionally conflicting, additional investigation is required to thoroughly assess the adverse reaction profile of EOs that have promising antidiabetic properties; such evaluations should also encompass the primary chemical ingredients of these oils.

## 5. Conclusions

EOs exert their hypoglycemic properties, which can be linked to their ability to increase glucose uptake, hinder glucose production, and improve INS sensitivity, as well as their antioxidant effects, through a wide range of mechanisms, including scavenging free radicals, regulating antioxidant enzymes, and decreasing lipid peroxidation. Due to the diverse chemical composition of phytocompounds that are found in certain plants, these effects emphasize the potential of EOs as adjuvant therapy in the management of both types of diabetes. However, more thorough clinical research is required to ascertain the ideal concentrations, safety profiles, and long-term impacts of EOs in the management of diabetes mellitus.

## Figures and Tables

**Table 1:** Results from clinical studies regarding the use of essential oils in the prevention/treatment of diabetes.

Description of the Study	Results/Observations	Ref.
*Nigella saliva* EO		
Clinical randomized study including 41 patients with T2DM consumed black cumin EO for 40 days (adjusting the daily dose so that it was the same as of the oil extracted from 0.7 g of *Nigella sativa* seeds), followed by a placebo for 40 more days. Blood samples were collected a jeun on days 0, 40, and 80 of the research, in the case of each subject.	After EO administration, a significant decrease in fasting glucose and an increase in AST and INS levels vs. baseline levels were found. INS and glucose levels reversed after the placebo period. Blood urea and ALT and the number of platelets and of leukocytes did not change in the two periods vs. the baseline values. It resulted that this EO may have a role in the treatment of T2DM, ensuring good hepatic and renal safety.	[103]
***Cuminum cyminum*** **L. EO**		
Clinical randomized double-blind study, 60 days, including 99 patients; 33 subjects were selected/included in each of the 3 groups: 1st group, one *C. cyminum* capsule (100 mg/day); 2nd group, one *C. cyminum* capsule each (50 mg/day); 3rd group placebo as control. A single blood sample was taken before/after 60 days of treatment.	HOMA-IR was considerably greater in the first two groups but reduced in the 3rd group. Mean of the FBS, HbA1c, and the serum levels of INS were significantly diminished. Upon completion of the study, in all the three groups, the mean serum levels of hsCRP and TNF-α were significantly decreased, and that of adiponectin was significantly higher.	[104]
Clinical randomized double-blind study, 90 days, including 95 T2DM patients distributed in each of the 3 groups: 1st group received capsule form of EO (25 mg/day); 2nd group, vitamin E (800 IU—150 mg); 3rd group, placebo (gelatin capsules) as control. A single blood sample was taken before/after 90 days of treatment.	First group had reduced values in blood glucose, HbA1c, ox-LDL, leptin, and triglyceride, ApoA1 and paraoxonase1 were increased. ApoA1, ApoB, blood glucose, HbA1c, leptin, lipid profile, oxLDL, and paraoxonase1 were determined, resulting decreasing in oxLDL, and significantly higher values for paraoxonase 1 in the second group at the end of the study. EO vs. vitamin E had stronger impact, being more efficient in diabetic index reduction.	[105]
Clinical randomized triple-blind trial, 56 days, 56 patients (between 18 and 60 years old), diagnosed with MetS, received 75 mg EO or placebo gelatin capsules 3 times a day. A single blood sample was taken before/after 56 days of treatment.	In patients with MetS, the results revealed that EO administration has an effect only on DBP among all MetS components.	[106]
Multi-center randomized, placebo-controlled, double-blind parallel-group clinical trial, including 54 pre-diabetic patients >19 years, distributed in each of the two groups: first group, that received capsule form of cumin EO (75 mg/day) 10 weeks vs. placebo as control.	Improved INS sensitivity (HOMA-IR, fasting serum INS, and QUICKI), lipid profile, and anthropometric parameters among individuals at risk for developing diabetes were observed following treatment with cumin EO. HbA1C, FPG, and leptin blood levels did not significantly improve, however.	[107]
Randomized double-blind placebo-controlled clinical, including 78 overweight subjects distributed in each of the 3 groups:: 1st group, one *C. cyminum* capsule (100 mg/day); 2nd group, orlistat120 capsule; 3rd group placebo as control (8 weeks).	When comparing orlistat, placebo, and *Cuminum cyminum* L., investigators found that QUICKI was significantly increased, while blood INS levels and HOMA-B were considerably decreased. Overweight patients who received *C. cyminum* L. experienced the same reductions in weight and body mass index as those who took orlistat120, also observing improvements in INS metabolism vs. placebo and orlistat120 group.	[108]

Alanine transaminase (ALT), apolipoprotein A1 (ApoA1), apolipoprotein B (ApoB), aspartate aminotransferase (AST), diastolic blood pressure (DBP), essential oil (EO), fasting blood sugar (FBS), glycated hemoglobin HbA1c, highly sensitive C-reactive protein (hsCRP), insulin (INS); insulin sensitivity (HOMA-IR), quantitative insulin sensitivity check index (QUICKI), MetS (metabolic syndrome), oxidized low-density lipoprotein (oxLDL), type 2 diabetes mellites (T2DM), tumor necrosis factor alpha (TNF-α).

## Data Availability

Data provided in this manuscript are supported by the inserted references.
